# Aberrant Methylation of 20 miRNA Genes Specifically Involved in Various Steps of Ovarian Carcinoma Spread: From Primary Tumors to Peritoneal Macroscopic Metastases

**DOI:** 10.3390/ijms23031300

**Published:** 2022-01-24

**Authors:** Vitaly I. Loginov, Irina V. Pronina, Elena A. Filippova, Alexey M. Burdennyy, Svetlana S. Lukina, Tatiana P. Kazubskaya, Leonid A. Uroshlev, Marina V. Fridman, Olga I. Brovkina, Natalya V. Apanovich, Alexander V. Karpukhin, Ivan S. Stilidi, Nikolay E. Kushlinskii, Alexey A. Dmitriev, Eleonora A. Braga

**Affiliations:** 1 Institute of General Pathology and Pathophysiology, 125315 Moscow, Russia; loginov7w@gmail.com (V.I.L.); zolly_sten@mail.ru (I.V.P.); p.lenyxa@yandex.ru (E.A.F.); burdennyy@gmail.com (A.M.B.); sveta_sergeevna349@mail.ru (S.S.L.); leoniduroshlev@gmail.com (L.A.U.); brov.olia@gmail.com (O.I.B.); 2 N.N. Blokhin National Medical Research Center of Oncology, 115478 Moscow, Russia; oncogen5@ronc.ru (T.P.K.); stylidi@yandex.ru (I.S.S.); kne3108@gmail.com (N.E.K.); 3 Vavilov Institute of General Genetics, Russian Academy of Sciences, 119991 Moscow, Russia; marina-free@mail.ru; 4 Research Center for Medical Genetics, 115478 Moscow, Russia; apanovichn81@mail.ru (N.V.A.); karpukhin@med-gen.ru (A.V.K.); 5 Engelhardt Institute of Molecular Biology, Russian Academy of Sciences, 119991 Moscow, Russia; alex_245@mail.ru

**Keywords:** ovarian carcinoma, metastatic primary tumors, peritoneal metastases, miRNA genes, DNA methylation, gene expression, overall survival

## Abstract

Our work aimed to differentiate 20 aberrantly methylated miRNA genes that participate at different stages of development and metastasis of ovarian carcinoma (OvCa) using methylation-specific qPCR in a representative set of clinical samples: 102 primary tumors without and with metastases (to lymph nodes, peritoneum, or distant organs) and 30 peritoneal macroscopic metastases (PMM). Thirteen miRNA genes (*MIR107*, *MIR124-2*, *MIR124-3*, *MIR125B-1*, *MIR127*, *MIR129-2*, *MIR130B*, *MIR132*, *MIR193A*, *MIR339*, *MIR34B/C*, *MIR9-1*, and *MIR9-3*) were hypermethylated already at the early stages of OvCa, while hypermethylation of *MIR1258*, *MIR137*, *MIR203A*, and *MIR375* was pronounced in metastatic tumors, and *MIR148A* showed high methylation levels specifically in PMM. We confirmed the significant relationship between methylation and expression levels for 11 out of 12 miRNAs analyzed by qRT-PCR. Moreover, expression levels of six miRNAs were significantly decreased in metastatic tumors in comparison with nonmetastatic ones, and downregulation of miR-203a-3p was the most significant. We revealed an inverse relationship between expression levels of miR-203a-3p and those of *ZEB1* and *ZEB2* genes, which are EMT drivers. We also identified three miRNA genes (*MIR148A*, *MIR9-1*, and *MIR193A*) that likely regulate EMT–MET reversion in the colonization of PMM. According to the Kaplan–Meier analysis, hypermethylation of several examined miRNA genes was associated with poorer overall survival of OvCa patients, and high methylation levels of *MIR130B* and *MIR9-1* were related to the greatest relative risk of death.

## 1. Introduction

Ovarian carcinoma (OvCa) is the most aggressive tumor among malignant neoplasms of the female reproductive system, and, in terms of mortality, it takes first place among oncogynecological diseases in the world [1,2]. OvCa is one of the hardest to detect cancers because it develops asymptomatically up to the advanced stages with extensive metastasis [3,4]. Extensive metastatic dissemination probably takes place because it occurs mainly in the peritoneum, which does not require tumor cells to overcome intravasation and extravasation. Although hematogenous and lymphogenous metastasis is also inherent in OvCa, peritoneal carcinomatosis dominates [5]. Early metastasis into the abdominal cavity with the flow of peritoneal fluid leads to the gradual formation of malignant ascites, which, along with tumor cells, contain tumor-associated immune and endothelial cells as well as tumor-associated fibroblasts [6]. All this creates a unique microenvironment that promotes the progression of OvCa, suppression of the immune system, and the development of chemotherapy resistance. The predominance of intraperitoneal metastasis makes the progression of OvCa unique, different from the classical and more studied hematogenous and lymphogenous metastasis, which is characteristic of most types of tumors [7,8].

Specific genes and signaling pathways, as well as epigenetic regulatory mechanisms, which include miRNAs, are involved in ensuring the pathogenesis and metastasis of OvCa [3,4]. MiRNAs play a critical role in cell cycle regulation, cell differentiation, proliferation, motility, adhesion, apoptosis, angiogenesis, stem cell function, stress response, and other fundamental biological processes associated with the development and progression of neoplasms. They can inhibit the expression of tumor-associated genes and function as both tumor suppressors and oncogenes. Numerous and convincing data on the role of miRNAs and their target genes in the pathogenesis and metastasis of OvCa were obtained [3,9,10,11].

It was noted that miRNA genes can be silenced by hypermethylation of promoter CpG islands, like protein-coding genes, and that the percentage of genes deregulated by aberrant methylation is significantly higher among miRNA genes than protein-coding genes [12]. We previously identified a group of hypermethylated miRNA genes in breast cancer and OvCa and showed their relationship with metastasis [13,14]. The present work was aimed at identifying miRNA genes specifically involved in the regulation of various stages of OvCa pathogenesis and metastasis. A detailed study of the methylation and expression levels of 20 miRNA genes in a representative set of 102 primary ovarian tumors and 30 peritoneal macroscopic metastases allowed us to identify a group of miRNA genes whose methylation was significantly associated with the onset of oncogenesis and specifically differentiate miRNAs involved in the regulation of metastasis at the stage of primary tumors and the stage of formation of secondary tumors (peritoneal macroscopic metastases) in OvCa.

## 2. Results

### 2.1. Aberrant Methylation of 20 miRNA Genes in the Development of Primary Tumors and Metastasis in Ovarian Cancer

**Figure 1:**
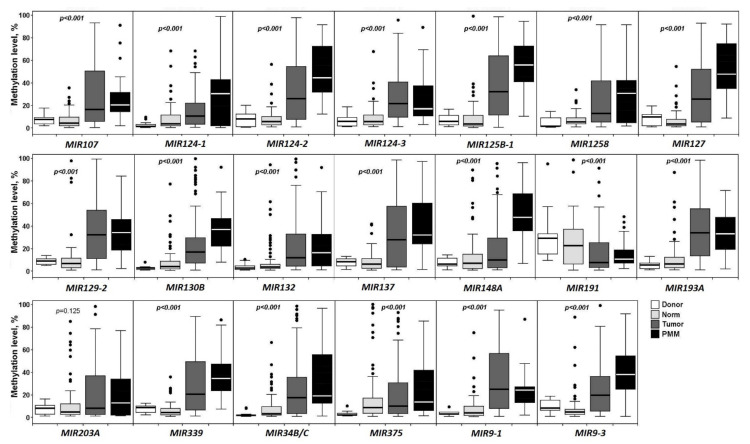
Methylation levels of 20 miRNA genes in 15 control samples (Donor), 83 histologically normal ovarian tissues of OvCa patients (Norm), 102 primary ovarian tumors (Tumor), and 30 peritoneal macroscopic metastases (PMM).

Methylation levels of 20 miRNA genes were evaluated in a representative set of 102 primary ovarian tumors (primary tumors of OvCa patients without and with metastases), 83 matched histologically normal ovarian tissues, 30 peritoneal macroscopic metastases, and 15 ovarian tissue samples from post-mortem “donors” without cancer in anamnesis. The results are presented in Figure 1.

As can be seen from Figure 1, a highly statistically significant (*p* < 0.001, FDR = 0.01) increase in the methylation level of 18 out of 20 miRNA genes was observed in OvCa passing from control samples (15 and 83 samples) to primary ovarian tumors (102) and peritoneal macroscopic metastases (30): *MIR107*, *MIR124-1*, *MIR124-2*, *MIR124-3*, *MIR125B-1*, *MIR1258*, *MIR127, MIR129-2, MIR130B, MIR132, MIR137, MIR148A, MIR193A, MIR339, MIR34B/C, MIR375, MIR9-1,* and *MIR9-3*, while the *MIR191* gene had a highly statistically significant (*p* < 0.001, FDR = 0.01) decrease in the methylation level. The methylation level increase of the *MIR203A* gene, considered in this way, turned out to be statistically insignificant.

The change in the methylation level of the majority of analyzed genes tended to be gradual from normal tissues to primary tumors and metastases. Special attention was drawn to the sharp increase in the methylation level of *MIR148A* during the transition from primary tumors to peritoneal macroscopic metastases (Figure 1).

**Figure 2:**
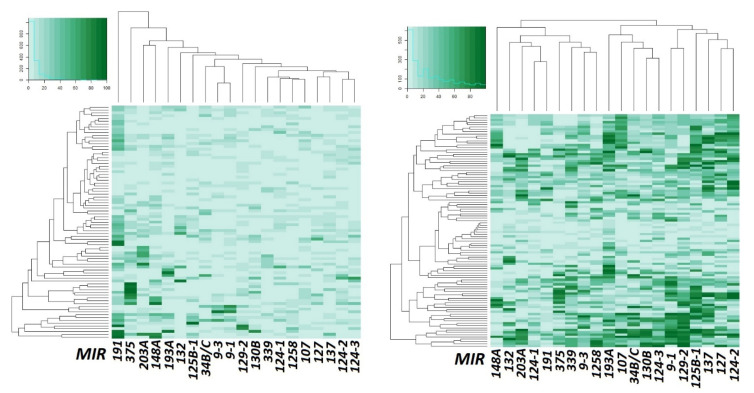
Heatmaps showing methylation levels of 20 miRNA genes in 83 samples of histologically normal ovarian tissues (**left**) and 102 samples of primary ovarian tumors (**right**). Samples are presented along the *Y*-axis, light green—low methylation level, dark green—high methylation level.

Data on the methylation level increase of most miRNA genes in primary ovarian tumors (102 samples) compared to matched histologically normal tissues (83) are also shown in Figure 2 in the form of heatmaps.

From an examination of Figure 2, it is quite obvious that the methylation level of most miRNA genes under study was strongly increased in primary ovarian tumors in comparison with matched histologically normal tissues.

**Figure 3:**
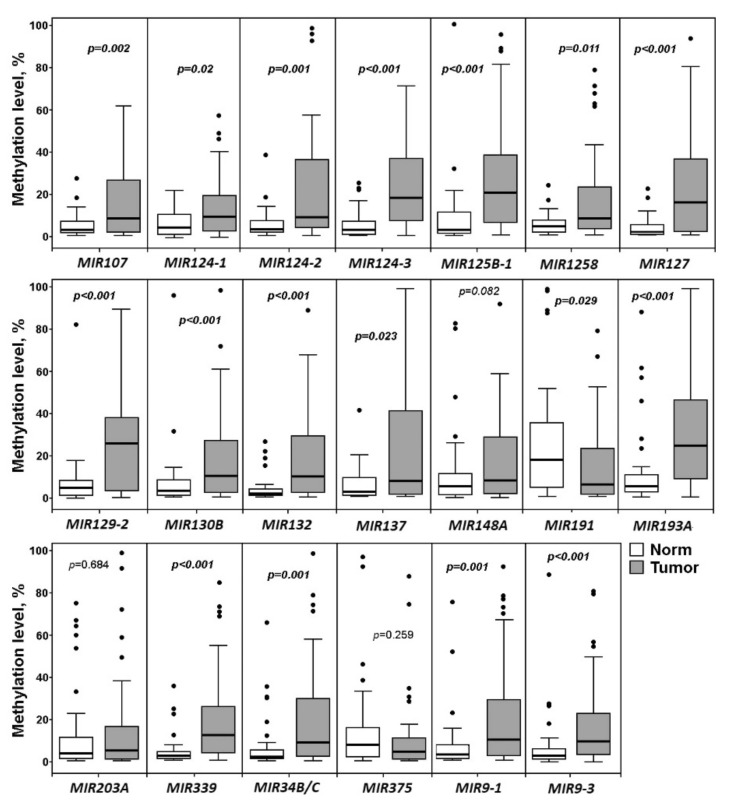
Methylation levels of 20 miRNA genes in 52 primary ovarian tumors of patients without metastases (Tumor) and 43 matched histologically normal ovarian tissues (Norm).

Next, we wanted to distinguish between miRNA genes associated with the onset of OvCa pathogenesis and those associated with the metastasis. First, we analyzed the data on methylation levels of 20 miRNA genes only in primary ovarian tumors of patients without metastases (52 samples) and matched histologically normal tissues (43) (Figure 3).

As can be seen from Figure 3, a highly statistically significant (*p* ≤ 0.002, FDR = 0.05) association with the onset of OvCa pathogenesis was shown for 13 out of 20 miRNA genes: *MIR107*, *MIR124-2*, *MIR124-3*, *MIR125B-1*, *MIR127*, *MIR129-2*, *MIR130B*, *MIR132*, *MIR193A*, *MIR339*, *MIR34B/C*, *MIR9-1*, and *MIR9-3*. Attention was drawn to the absence of a statistically significant relationship with the early (premetastatic) stages of OvCa development for three genes: *MIR148A*, *MIR203A*, and *MIR375* (Figure 3). For four miRNA genes (*MIR124-1*, *MIR1258*, *MIR137*, and *MIR191*), the association with the early stages was much less statistically significant (0.002 < *p* < 0.05) than with the OvCa pathogenesis as a whole (compare Figure 1 and Figure 3).

**Figure 4:**
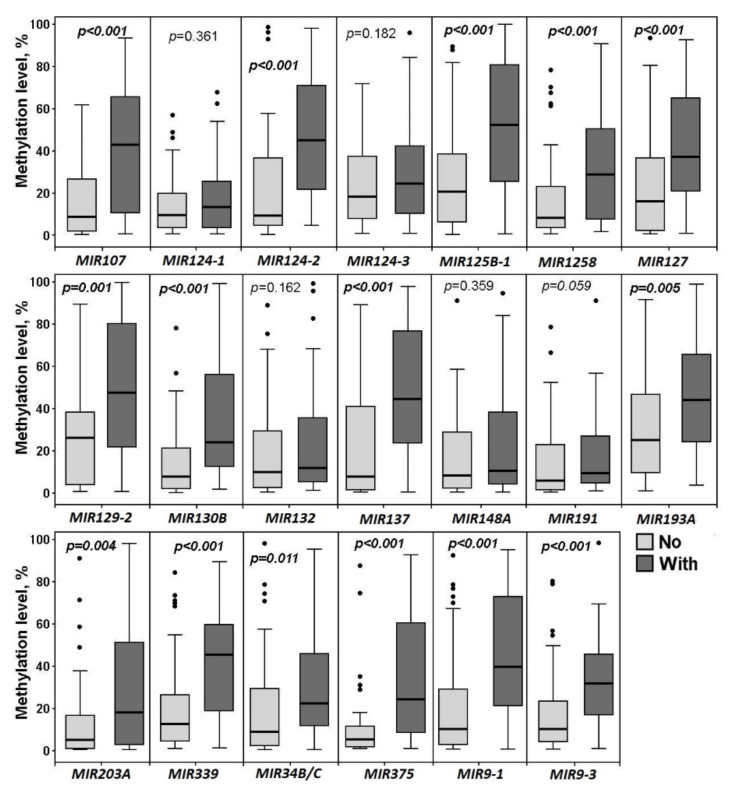
Methylation levels of 20 miRNA genes in 52 primary ovarian tumors of patients without metastases (No) and 50 primary ovarian tumors of patients with metastases (With).

Next, it was of interest to study whether there are specific differences in methylation levels of any of the miRNA genes under study between primary tumors of patients without metastases (52 samples) and those of patients with metastases (50). So, we compared these two groups and the results of the analysis can be found in Figure 4.

The increase in the methylation level of 14 out of 20 miRNA genes (*MIR107*, *MIR124-2*, *MIR125B-1*, *MIR1258*, *MIR127*, *MIR129-2*, *MIR130B*, *MIR137*, *MIR193A*, *MIR203A*, *MIR339*, *MIR375*, *MIR9-1*, and *MIR9-3*) was highly associated (*p* ≤ 0.005, FDR = 0.05) with metastatic primary ovarian tumors (Figure 4). It is interesting to note that high methylation levels of *MIR1258*, *MIR137*, *MIR203A*, and *MIR375* were almost exclusively revealed in metastatic primary tumors and practically not observed at the early stages of OvCa pathogenesis. We nearly did not detect methylation of the *MIR148A* gene in 102 primary tumors (both metastatic and nonmetastatic), however, this gene was highly hypermethylated in peritoneal macroscopic metastases (Figure 1, Figure 2, Figure 3 and Figure 4).

Thus, methylation levels of *MIR1258*, *MIR137*, *MIR203A*, and *MIR375* may be suggested as specific biomarkers of OvCa dissemination; and *MIR148A—*as a specific biomarker of peritoneal macroscopic metastases.

### 2.2. Relationship of Methylation of Examined miRNA Genes with the Clinical Stage and Histological Grade of Ovarian Cancer

**Figure 5:**
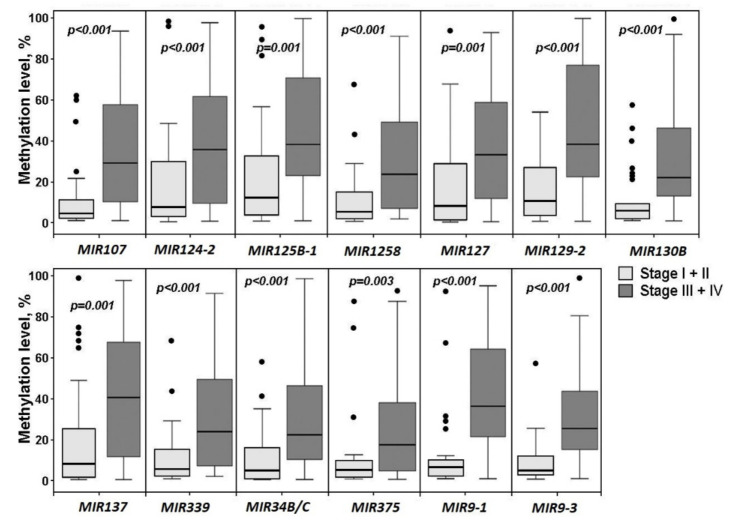
Hypermethylated miRNA genes associated with advanced clinical stages of OvCa. The set of OvCa samples included 29 samples of stages I + II and 73 samples of stages III + IV.

Methylation levels of 20 miRNA genes were compared between the early stages of OvCa and the late ones. Hypermethylation of 13 miRNA genes was shown to be associated with more advanced stages (III + IV versus I + II) of OvCa (Figure 5). Comparison of a group of 14 miRNA genes whose methylation in primary tumors was associated with metastasis (Figure 4) and a group of 13 genes whose methylation was associated with advanced stages (Figure 5) revealed an overlap of 12 common genes: *MIR107*, *MIR124-2*, *MIR125B-1*, *MIR1258*, *MIR127*, *MIR129-2*, *MIR130B*, *MIR137*, *MIR339*, *MIR375*, *MIR9-1*, and *MIR9-3*. A specific difference was identified for three genes: methylation of *MIR193A* and *MIR203A* was less statistically significantly (*p* = 0.038 and *p* = 0.099 respectively) associated with advanced clinical stages and, on the contrary, methylation of *MIR34B/C* was highly statistically significantly (*p* < 0.001, FDR = 0.01) associated with stages III/IV of OvCa (compare Figure 4 and Figure 5).

It is worth emphasizing that the overlap of 12 genes indicated the importance of common genes and common processes in the metastasis of primary tumors and the progression of the disease stage.

**Figure 6:**
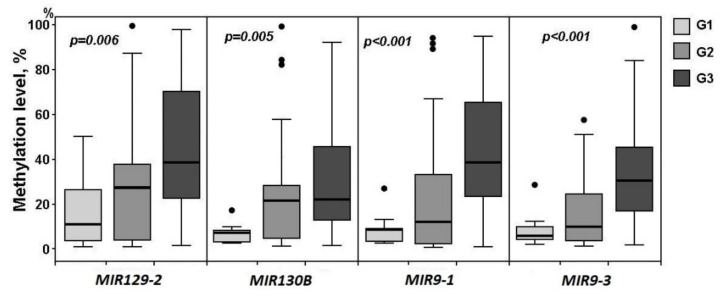
Hypermethylated miRNA genes associated with ovarian tumor grade. The sample set included: G1—9 samples, G2—27 samples, and G3—59 samples.

Further, we searched for miRNA genes whose methylation level changes were significantly associated with the histological grade of primary ovarian tumors. Only four genes (*MIR129-2*, *MIR130B*, *MIR9-1*, and *MIR9-3*) were found to be statistically significantly (*p* ≤ 0.006, FDR = 0.05) associated with the OvCa histological grade, judging by the level of their methylation (Figure 6).

Thus, among 14 miRNA genes involved in the processes of cell metastasis in primary ovarian tumors, 12 genes were also associated with an advanced clinical stage and only four genes with a histological grade.

### 2.3. Hypermethylated miRNA Genes Specifically Involved in the Formation of Macroscopic Metastases in the Peritoneum of Ovarian Cancer Patients

**Figure 7:**
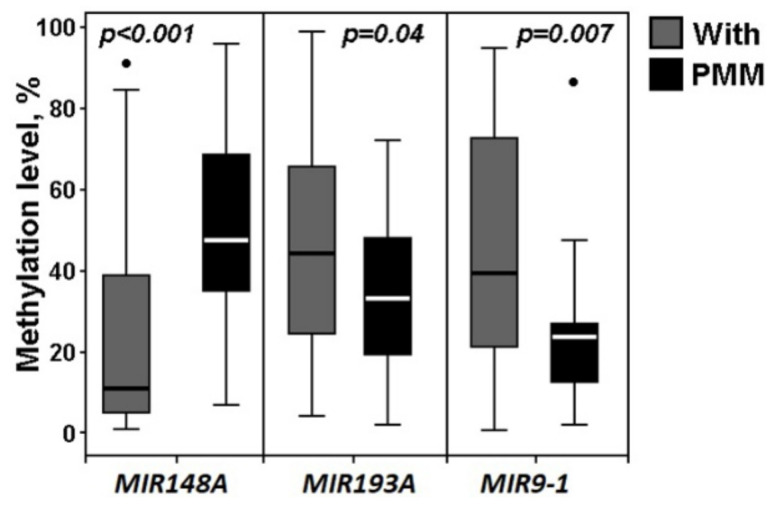
Methylation levels of miRNA genes in 30 peritoneal macroscopic metastases (PMM) and 50 primary tumors of OvCa patients with metastases (With).

We paid special attention to the effect of hypermethylated miRNA genes on the transition from primary metastatic tumors to formed macroscopic peritoneal metastases (Figure 7).

As follows from Figure 7, the formation of peritoneal macroscopic metastases was highly associated with hypermethylation of the *MIR148A* gene, while this gene did not increase the methylation level either at the early premetastatic stages of OvCa development or at the stages of the metastatic process in primary ovarian tumors (compare with Figure 1, Figure 2, Figure 3 and Figure 4). It is noteworthy that methylation of the *MIR148A* gene did not accompany either an increase in the clinical stage of OvCa or an increase in the histological grade (compare with Figure 5 and Figure 6). It can be concluded that hypermethylation of the *MIR148A* gene is a highly significant factor in the colonization of peritoneal metastases. Moreover, among the 20 studied miRNA genes, hypermethylation of the *MIR148A* gene was the only factor that highly statistically significantly (*p* < 0.001, FDR = 0.01) promoted the formation of secondary tumors.

In addition, in peritoneal metastases, we observed a significant decrease in methylation levels of *MIR193A* (*p* = 0.04) and *MIR9-1* (*p* = 0.007, FDR = 0.05), which were significantly hypermethylated both at the stage of the onset of primary ovarian tumors and at the stage of their metastasis. This phenomenon may be associated with a partial reversal of epithelial–mesenchymal transition (EMT) into mesenchymal–epithelial transition (MET) in secondary tumors, which may be associated with the loss of methylation by these genes.

### 2.4. The Functional Significance of Methylation of Examined miRNA Genes in the Regulation of Their Expression in Ovarian Cancer

**Figure 8:**
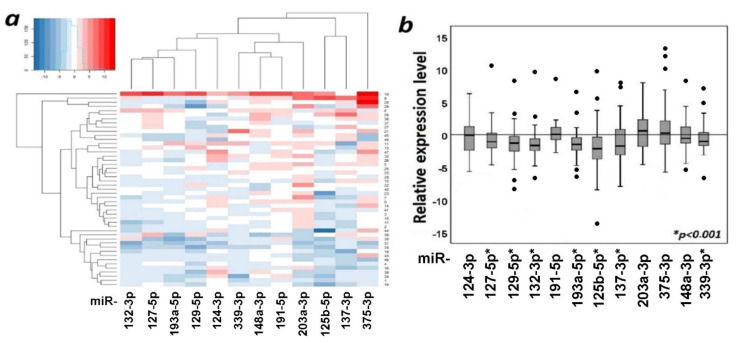
Changes in the levels of 12 miRNAs in 47 primary tumors relative to matched histologically normal tissues; (**a**) heatmap (blue color—decrease in expression, red color—increase in expression); (**b**) expression box plots and the significance of the changes.

The functional role of aberrant methylation of miRNA genes was tested by the effect of methylation on the expression of 12 miRNAs in 47 paired (tumor/normal) ovarian samples. The data on changes in the levels of 12 miRNAs in primary tumor samples are shown in Figure 8.

As can be seen from Figure 8, seven miRNAs statistically significantly (*p* < 0.001, FDR = 0.01) reduced expression in primary tumors, namely, miR-125b-5p, miR-127-5p, miR-129-5p, miR-132-3p, miR-137-3p, miR-193a-5p, and miR-339-3p. These data were in good agreement with the data on hypermethylation of genes encoding these miRNAs in primary ovarian tumors.

**Figure 9:**
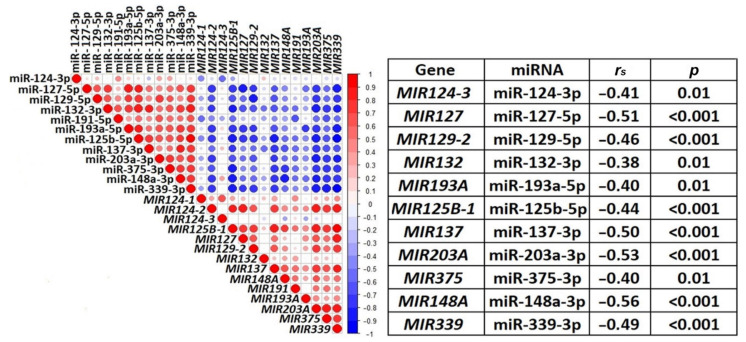
Significant negative correlation between the levels of methylation and expression for 11 miRNA genes in primary ovarian tumors (47 paired samples). The correlation plot is shown on the left (blue circles—negative correlation, red circles—positive correlation, size and brightness of circles represent the degree of correlation). On the right, Spearman’s correlation coefficients (*r_s_*) and their statistical significance (*p*) are shown for pairs of aberrantly methylated miRNA genes and miRNAs with altered expression levels.

Furthermore, the relationship between methylation and expression of 12 miRNAs was characterized using a correlation plot and Spearman’s correlation coefficient (*r_s_*, Figure 9). Only for miR-191-5p, the correlation of expression levels with methylation levels was not statistically significant.

As can be seen from Figure 9, a statistically significant negative correlation (*r_s_* was in the range from −0.38 to −0.56, *p* ≤ 0.01, FDR = 0.05) between levels of methylation and expression was shown for 11 miRNAs (miR-124-3p, miR-125b-5p, miR-127-5p, miR-129-5p, miR-132-3p, miR-137-3p, miR-148a-3p, miR-193a-5p, miR-203a-3p, miR-339-3p, and miR-375-3p), and highly statistically significant negative correlation (*r_s_* was in the range from −0.44 to −0.56, *p* < 0.001, FDR = 0.01) for 7 of them (miR-125b-5p, miR-127-5p, miR-129-5p, miR-137-3p, miR-148a-3p, miR-203a-3p, and miR-339-3p). This result indicated the functional significance of hypermethylation in downregulation of miRNA genes in OvCa.

### 2.5. Comparison of Expression Levels of 12 miRNAs in Primary Tumors of Ovarian Cancer Patients without and with Metastases and in Macroscopic Peritoneal Metastases

**Figure 10:**
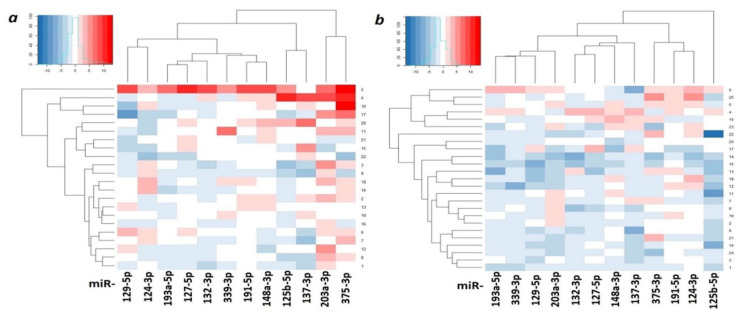
Heatmaps of relative expression levels of 12 miRNAs in nonmetastatic primary ovarian tumors ((**a**), 22 samples, tumor/norm) and metastatic ones ((**b**), 25 samples, tumor/norm). Samples are presented along the *Y*-axis, blue—expression decrease, red—expression increase.

Heatmaps were constructed, reflecting the expression levels of 12 miRNAs in primary tumors of OvCa patients without and with metastases relative to matched histologically normal tissues (Figure 10).

Heatmaps (Figure 10) clearly showed a pronounced decrease in expression in most studied miRNAs in primary tumors of patients with metastatic OvCa.

**Figure 11:**
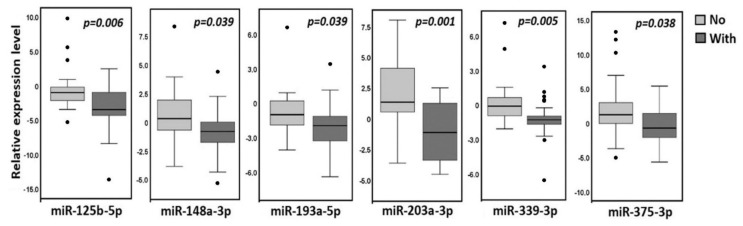
The effect of metastasis on the miRNA expression level in primary tumors of OvCa patients. Data for six miRNAs with a statistically significant decrease in the expression level in 25 samples of primary tumors of patients with metastases (With) in comparison with 22 samples of primary tumors of patients without metastases (No) are shown.

As can be seen from Figure 11, there was a statistically significant (*p* < 0.05) decrease in the expression level of six miRNAs in metastatic primary tumors in comparison with nonmetastatic ones. Moreover, the decrease was highly statistically significant for miR-125b-5p and miR-339-3p (*p* ≤ 0.006, FDR = 0.05) and the most statistically significant for miR-203a-3p (*p* = 0.001, FDR = 0.01).

The strongest decrease in miR-203a-3p expression in primary tumors of patients with metastases corresponded to a sharp appearance of hypermethylation of the *MIR203A* gene exclusively in primary tumors of patients with metastases. Thus, hypermethylation of the *MIR203A* gene may be a very useful biomarker of metastatic primary ovarian tumors.

The analysis of expression levels of 12 miRNAs in nine peritoneal macroscopic metastases relative to nine matched samples of primary tumors was carried out (Table 1).

We noted a twofold drop in the median expression level of miR-148a-3p (from –0.70 to –1.45) that was consistent with the data on a sharp increase in the methylation level of the *MIR148A* gene during the transition from primary metastatic tumors to peritoneal macroscopic metastases (see Figure 7). Although the analysis of expression was not statistically confirmed owing to the small set of samples, it is possible to suggest a specific role for inactivating methylation of the *MIR148A* gene in the formation of secondary tumors. In addition, the decrease in the methylation level of two miRNA genes, *MIR193A* (*p* = 0.04) and *MIR9-1* (*p* = 0.007, FDR = 0.05), which were hypermethylated in primary tumors, was found in secondary tumors that may suggest the involvement of *MIR148A, MIR193A*, and *MIR9-1* genes in the regulation of partial reversion EMT-MET during colonization of secondary ovarian tumors in the peritoneum.

### 2.6. Reverse Relationship between Expression Levels of miRNA-203a-3p and ZEB1 and ZEB2 Genes in Ovarian Cancer

Data on the significant hypermethylation of the *MIR203A* gene in primary ovarian metastatic tumors and almost complete absence of *MIR203A* methylation in tumors of OvCa patients without metastases, which were in full agreement with the most significant decrease in the expression level of this gene in metastatic primary tumors, indicated the role of this miRNA in the initial stage of metastasis. This result was consistent with the data on the participation of miR-203a-3p in suppression of the EMT in OvCa cell cultures SKOV3 and OVCAR3 and the Xenograft mouse model [15].

As known, the hallmarks of EMT in cancer are the upregulation of N-cadherin and the downregulation of E-cadherin. Transcription factors ZEB1 (Zinc finger E-box binding homeobox 1) and ZEB2 (Zinc finger E-box binding homeobox 2) specifically bind to multiple enhancer boxes (E-boxes) located within the transcription regulatory regions of the E-cadherin gene, and their interaction inhibits E-cadherin expression and, in this way, *ZEB1* and *ZEB2* genes serve as key drivers of EMT in multiple human cancers [16,17].

According to the TargetScan 7.2 database, both sites of *ZEB1* and *ZEB2* that matched miR-203a-3p represented 8-mer in conserved 3′-untranslated regions (3′-UTRs) of the genes. Such locations of base pairing indicated the inhibitory properties of miR-203a-3p for target gene expression. From 608 cases of OvCa in The Cancer Genome Atlas database (TCGA-OV data set), we selected 583 cases with primary ovarian tumors (35% were from patients with metastases, this number was close to the histological characteristics of our cohort) and compared expression levels of *ZEB1*, *ZEB2*, and miR-203a-3p in this sample set with their expression levels in normal samples from the GEO database. Expression of both *ZEB1* and *ZEB2* was differential in OvCa samples (TCGA vs. GEO, *p* < 0.05) and present in all analyzed tumor and normal tissues. MiR-203a-3p expression was dysregulated (*p* < 0.05) and was detected in 98% of tumor samples and 97% of normal ovarian tissue samples.

**Figure 12:**
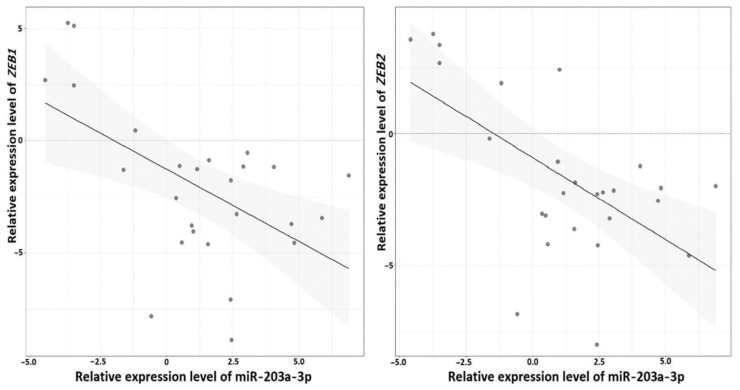
Negative correlation between the levels of miR-203a-3p and mRNA of *ZEB1* and *ZEB2* (26 paired samples were analyzed, marked in Supplementary Table S1).

To define the association between miR-203a-3p and the *ZEB1* and *ZEB2* genes, we performed expression analysis in a subset of 26 paired OvCa samples (all samples that were available at the time of this part of the study) using qPCR and calculated correlation coefficients between expression levels for two miRNA–mRNA pairs (Figure 12).

Spearman’s correlation coefficients for both pairs, *ZEB1*/miR-203a-3p and *ZEB2*/miR-203a-3p, were negative: –0.41 and –0.45 (*p* = 0.04 and 0.02 respectively). These data suggested direct or indirect interactions of miR-203a-3p with mRNA of *ZEB1* and *ZEB2* genes, and further supported the involvement of miR-203a-3p in EMT.

### 2.7. Effect of Aberrant Methylation of Examined miRNA Genes on the Survival of Patients with Ovarian Cancer

To assess the clinical significance of 20 aberrantly methylated miRNA genes, the relationship between their methylation levels and patient survival was studied. The results of the ROC analysis are shown in Table 2.

**Figure 13:**
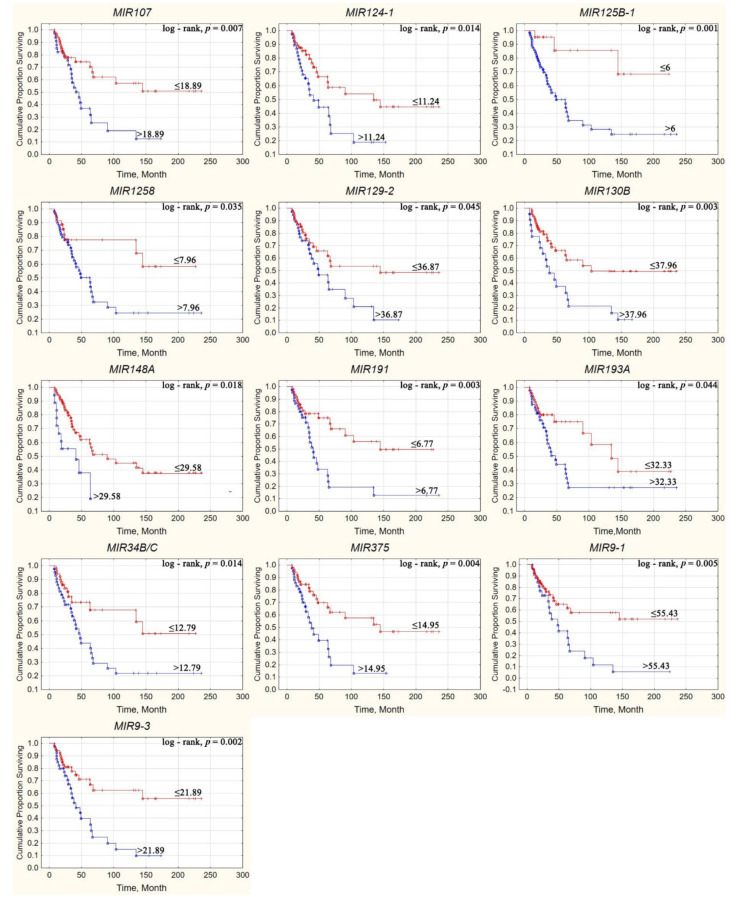
The Kaplan–Meier curves with miRNA gene methylation higher/lower cutoff values.

Using the ROC analysis, seven hypermethylated miRNA genes (*MIR107*, *MIR124-3*, *MIR125B-1*, *MIR130B*, *MIR34B/C*, *MIR9-1*, and *MIR9-3*) were identified as significantly associated with shorter overall survival in OvCa patients. Using the cutoff values in Table 2, it was possible to construct Kaplan–Meier curves for evaluation of patient survival under different methylation levels of the miRNA genes. A comparison of Kaplan–Meier curves with miRNA gene methylation above/below cutoff values using a log-rank test demonstrated an association of overall survival with the methylation of 13 miRNA genes (Figure 13).

Thus, according to the Kaplan–Meier analysis, methylation levels of 13 miRNA genes (*MIR107*, *MIR124-1*, *MIR125B-1*, *MIR1258*, *MIR129-2*, *MIR130B*, *MIR148A*, *MIR191*, *MIR193A*, *MIR34B/C*, *MIR375*, *MIR9-1*, and *MIR9-3*) were significantly associated with the survival of patients with OvCa. Fisher’s exact test and Cox proportional hazards model were additionally used to identify the miRNA genes most reliably associated with survival and to obtain their quantitative estimates. The results are presented in Table 3.

Analysis of the obtained results allowed us to determine genes significantly associated with patient survival according to all criteria used: *MIR107*, *MIR125B-1*, *MIR130B*, *MIR9-1*, and *MIR9-3*. Among them, hypermethylation of *MIR130B* and *MIR9-1* genes led to the highest relative risk of death.

## 3. Discussion

This work identified aberrantly methylated miRNA genes specifically associated with different steps of OvCa pathogenesis and metastatic spread. As mentioned above, in addition to hematogenous and lymphogenous metastasis, in OvCa, dissemination of metastases to the peritoneum is observed, which is recognized as dominant in this disease [5,6,7,8]. In connection with these data, methylation levels of 20 miRNA genes were characterized in a representative set of 102 primary tumors, including 52 tumors of patients without metastases and 50 tumors of patients with metastases, as well as 30 macroscopic peritoneal metastases.

As a main result, it was shown that different groups of miRNAs were involved in different stages of OvCa pathogenesis and metastatic spread. Among them, *MIR1258*, *MIR137*, *MIR203A*, and *MIR375* were the most specific. Hypermethylation of these four miRNA genes was associated with the onset of the metastatic process in primary tumors, while hypermethylation of the *MIR148A* gene was specifically associated with the formation of secondary tumors in the peritoneum.

In a subset of 47 paired OvCa samples, a strong correlation between alterations of methylation and expression levels for 11 out of 12 miRNAs examined by qRT-PCR (miR-124-3p, -125b-5p, -127-5p, -129-5p, -132-3p, -137-3p, -148a-3p, -193a-5p, -203a-3p, -339-3p, and -375-3p) was established that indicated the functional significance of hypermethylation in the downregulation of these miRNA genes in OvCa. At the same time, for six genes, a relationship was shown between a decrease in their expression level in primary tumors and metastasis, which confirmed the tumor suppressor role and antimetastatic function for a number of miRNAs studied. Downregulation in these tumors was the most statistically significant for miR-203a-3p (*p* = 0.001, FDR = 0.01), which was distinguished by specific hypermethylation only in the patients with metastases. These data suggested that hypermethylation of the *MIR203A* gene can be used as a marker predicting the development of metastases in OvCa.

One of the critical stages of metastasis is the epithelial–mesenchymal transition (EMT), which is involved in different stages of the metastatic process but primarily provokes migration and invasion of tumor cells and triggers metastasis [18,19]. Genes of miRNAs involved in the metastasis of primary tumors may be involved in EMT. In this regard, we determined in silico the relationship between the expression levels of miR-203a-3p and those of the *ZEB1* and *ZEB2* genes, widely known as key factors of EMT in cancer, including OvCa, and inhibitors of E-cadherin, which is responsible for maintaining the epithelial phenotype of cells [20,21,22,23].

The sequence of miR-203a-3p is complementary to conservative sites of 3′-UTRs of *ZEB1* and *ZEB2* that suggests miR-203a-3p to be a tumor suppressor. Furthermore, the inhibitory effect of miR-203a-3p on these target genes is supported by the significant negative correlation between the expression levels of miR-203a-3p and mRNA of *ZEB1* and *ZEB2*. The effect of miR-203 (the commonly used name of miR-203a-3p) on *ZEB1* and *ZEB2* genes was previously shown in many cancer types, such as lung, gastric, cervical, prostate, colon, and renal cancers, as well as glioblastoma, cholangiocarcinoma, and nasopharyngeal carcinoma, for both *ZEB1* [24,25,26,27,28] and *ZEB2* [29,30,31,32,33,34]. However, the inhibitory effect of miR-203a-3p/miR-203 on the expression level of *ZEB1* and *ZEB2* in OvCa was not previously reported in the literature (PubMed on 15 November 2021).

Our results on the negative correlation of the miR-203a-3p level with mRNA levels of *ZEB1* and *ZEB2* in OvCa indicated the role of miR-203a-3p in suppressing EMT through inhibitory interaction with these protein-coding genes, key activators of EMT. The identified interactions of miR-203a-3p with mRNA of the *ZEB1* and *ZEB2* genes in OvCa can occur through direct binding and indirectly through chains of interactions. However, direct linking is quite likely because data on direct interactions of miR-203 with *ZEB1* and *ZEB2* in other cancer types were obtained, as cited above. Moreover, one site for binding miR-203 was bioinformatically localized in 3′-UTR of *ZEB1* [25] and more than one site in 3′-UTR of *ZEB2* [32]. It was noted that interactions of miR-203 with *ZEB1* and *ZEB2* suppressed metastasis and EMT in multiple cancers and, in addition, antimetastatic miR-203, inhibiting *ZEB2*, reduced tumor stemness and chemotherapy resistance, for example, in nasopharyngeal carcinoma [31].

In 2011, Hanahan and Weinberg, analyzing biological manifestations in the process of multistep development and progression of human tumors, included reprogramming of tumor cells as an additional hallmark [35]. In the following reviews, Weinberg et al. highlighted changes in transcription programs in the process of multistage tumorigenesis and metastasis, as well as the role of epigenetic factors in the regulation of reprogramming processes, including EMT and plasticity in cancer [18,36,37,38]. Moreover, there are points of view that the mesenchymal–epithelial transition (MET) occurs in order to fix metastatic cells in the metastatic niche, and the reversibility of EMT–MET provides plasticity for all biological processes during cancer metastasis [39]. For example, there has been a plastic reversion of EMT–MET during colonization of distant metastases of colon cancer in the liver [33]. OvCa metastasis into the peritoneum is particularly flexible, it is assumed that EMT–MET reversion is incomplete, can turn back, and the features of both epithelial and mesenchymal cells are preserved [19,40,41].

In these processes, the regulatory function can be performed by epigenetic factors [36,42]. In line with this concept, we observed that an increase in the methylation levels of the *MIR193A* and *MIR9-1* genes in primary tumors was replaced by a partial decrease in methylation levels in peritoneal macroscopic metastasis that can partially reactivate the expression of miR-193a and miR-9 in these secondary tumors. Thus, we speculated a partial reversal of EMT-MET with the acquisition by cells of secondary tumors of epithelial properties and suggested a regulatory role in this reversal for miR-193a and miR-9 owing to partial demethylation of genes encoding these miRNAs. This concept is also consistent with our recently discovered effect of decreasing hypermethylation of a number of long noncoding RNA genes during the transition from primary ovarian tumors to peritoneal macroscopic metastases [43]. The features we discovered corresponded to the concept of cancer plasticity, including EMT–MET and their transitional forms as pEMT (partial EMT), or a multistep dynamic continuous spectrum of plastic transition EMT–MET with metastable intermediate states [19,44,45].

In addition, the relationship between miRNA gene methylation levels and overall survival of OvCa patients was established using the Kaplan–Meier analysis, Fisher’s exact test, and Cox proportional hazards model. The five genes, for which hypermethylation was the most strictly associated with poorer overall survival of OvCa patients, were selected: *MIR107*, *MIR125B-1*, *MIR130B*, *MIR9-1*, and *MIR9-3*. Worth noting, these five genes were a part of a group of 12 genes associated with both metastatic primary tumors and advanced stages. Among them, hypermethylation of *MIR130B* and *MIR9-1* genes led to the greatest relative risk of death.

Our results on the relationship between hypermethylation of *MIR9-1* and *MIR9-3* with a decrease in survival were consistent with the data on the effect of these miRNAs on paclitaxel resistance in OvCa cells [46]. Downregulation of miR-130b, including that due to the gene hypermethylation, increased the multidrug resistance of OvCa cells [47,48]. Increased resistance to therapy can result in decreased survival rates. A decrease in serum miR-125b levels was also significantly associated with an increase in chemoresistance in patients [49] that is consistent with our data on the relationship of *MIR125B-1* methylation with worse survival in OvCa patients. In addition, miR-125b encapsulated in hyaluronic acid-based nanoparticles (HA-PEI-miR-125b) in combination with intraperitoneal paclitaxel can enhance the antitumor efficacy of paclitaxel in patients with OvCa [50]. Moreover, miR-125b-nanoparticles did not induce systemic toxicity and therefore are promising in intraperitoneal chemotherapy of OvCa patients [50]. For miR-107, only two recent studies provided data on biological functions in OvCa. The study by Tang et al. [51] showed the ability of miR-107 to suppress proliferation and induce cell cycle arrest by targeting mRNA of *CCNE1* (encodes cyclin E1), and the work by Liu et al. [52] revealed that miR-107 inhibited cell proliferation, migration, and invasion in OvCa via targeting pyruvate dehydrogenase kinase isozyme 4 (PDK4). There were no data on the role of miR-107 in metastasis and on the clinical significance of this miRNA. We previously showed the role of the hypermethylated *MIR107* gene in the development and metastasis of OvCa [53], and in the present work, we were the first to obtain data on the significant effect of *MIR107* hypermethylation on a decrease in the survival rate of OvCa patients.

Our data on the specific participation of hypermethylated *MIR1258*, *MIR137*, *MIR203A*, *MIR375*, and *MIR148A* in several steps of OvCa spread strengthen the literature data on the role of miRNAs encoded by these genes in the progression, EMT, and metastasis of OvCa and other cancers. So, miR-1258 acted as a tumor suppressor to inhibit invasion and metastasis by targeting HPSE (heparanase) in gastric cancer and inhibited growth and EMT via targeting SP1 in oral squamous cell carcinoma [54,55]. However, data on the association of *MIR1258* with EMT and metastasis in OvCa were not reported by other authors. MiR-137 was shown to directly target Snail and inhibit EMT in OvCa, which is an early and critical step in metastasis [56]. MiR-375 was also demonstrated to inhibit the growth, metastasis, and drug sensitivity of OvCa cells [57]. The decreased expression level of miR-148a in OvCa was associated with lymph node metastasis and predicted poor prognosis [58]. In breast cancer, data were obtained on the participation of miR-148a in the suppression of the process of extravasation of cancer cells [59], which is in agreement with our data on the specific function of miR-148a in the colonization of macroscopic metastases of OvCa in the peritoneum.

It should be emphasized that in the works published by other authors on the role of miRNAs in OvCa, attention was focused on the analysis of miRNA expression levels and functional experiments in cell cultures. In our work, using a representative set of clinical samples, we demonstrated the significance of DNA methylation of a large group of 20 miRNA genes in biological processes at different stages of development and metastasis of OvCa and in the formation of peritoneal secondary tumors for the first time. Together, our results revealed aberrantly methylated miRNA genes as epigenetic factors specifically involved in the regulation of various steps of OvCa pathogenesis and metastasis and suggested novel potential biomarkers.

## 4. Materials and Methods

### 4.1. Tissue Samples

The set of samples included 102 primary ovarian tumors (primary tumors of OvCa patients without and with metastases), 83 matched histologically normal ovarian tissues, 30 peritoneal metastases, and 15 control samples (donors, which were ovarian tissues from deceased individuals without any cancer in their anamnesis). The characteristics of the samples are presented in Table 4 and Supplementary Table S1. The whole set of samples was used in the DNA methylation studies. A subset of 47 paired (tumor/normal) ovarian samples was used for the analysis of miRNA expression. The mRNA levels of *ZEB1* and *ZEB2* genes were evaluated in 26 paired OvCa samples remaining after the miRNA expression studies. All samples were obtained from the N.N. Blokhin National Medical Research Center of Oncology (Moscow, Russia). Most samples (77%, 79/102) were serous ovarian adenocarcinomas. The study was performed in accordance with the principles outlined in the Declaration of Helsinki. The samples were collected in accordance with the guidelines issued by the Ethics Committee of the N.N. Blokhin National Medical Research Center of Oncology and in cooperation with them. Tumor tissues and matched histologically normal tissues were obtained from the patients after surgical resection prior to radiation or chemotherapy and were stored in liquid nitrogen. Diagnoses were verified by histopathology, and only the samples containing 70–80% or more tumor cells were used in the studies. Matched controls were histologically confirmed to be normal epithelial cells. The tumor samples were characterized based on the tumor–node–metastasis according to the International System of Classification of Tumors, according to the staging classification of the Union for International Cancer Control [60], and using the criteria for classification developed by the World Health Organization (WHO) [61].

### 4.2. Bioinformatics

Protein-coding genes *ZEB1* and *ZEB2* were confirmed as potential targets for miR-203a-3p using the TargetScan 7.2 (http://www.targetscan.org/, accessed on 15 November 2021) and miRDB 6.2 (http://mirdb.org, accessed on 15 November 2021) databases. The next step was the assessment of Spearman’s correlation coefficient and significance level for two miRNA-mRNA pairs. For this purpose, we extracted counts of reads that overlap at our regions of interest from the TCGA-OV project (study accession phs000178, https://portal.gdc.cancer.gov/projects/TCGA-OV, accessed on 15 November 2021) as a tumor group and from GEO (accession numbers GSE14407, GSE18520, and GSE36668, https://www.ncbi.nlm.nih.gov/geo/, accessed on 15 November 2021) as a normal group. The choice of data sets was determined by the requirement of the presence of expression of miR-203a-3p and its target genes in more than 70% of samples. Using the removeBatchEffect procedure from the limma package R-studio, we eliminated systematic errors that occurred during the sequence alignment stage. The counts in data sets were normalized by FPKM (Fragments Per Kilobase Million) values. The Spearman’s correlation coefficients and significance levels were calculated with R 3.4.4.

### 4.3. DNA and Total RNA Isolation and Reverse Transcription

Nitrogen-frozen tissues were crushed using an Ultra-Turrax T10 basic homogenizer-dispersant (IKA, Staufen, Germany). DNA from the tissues was isolated using phenol extraction as per standard protocols. The quality and concentration of DNA were determined by the optical density on the NanoDrop ND-1000 spectrophotometer (Thermo Fisher Scientific, Waltham, MA, USA). Total RNA was isolated using a guanidinium thiocyanate-phenol-chloroform extraction protocol [62]. Before use, all RNA samples were treated with RNase-free DNase I (Thermo Fisher Scientific) according to the manufacturer’s protocol. The quality and concentration of RNA were evaluated using the NanoDrop ND-1000 spectrophotometer (Thermo Fisher Scientific). 28S and 18S rRNA band intensities were estimated via electrophoresis in a 2% agarose gel using the Sub-Cell GT Horizontal Electrophoresis System (Bio-Rad, Hercules, CA, USA) followed by gel imaging with the Gel Doc XR+ Gel Documentation System (Bio-Rad). The RNA was considered acceptable for further use if the bands of the 28S and 18S rRNAs had an intensity ratio of about 2:1, and the A260/A280 ratio was in the range 1.8–2.1. All cDNA was synthesized from 1 μg of total RNA using the MMLV reverse transcriptase and random specific set of oligonucleotides according to the manufacturer’s protocol (Thermo Fisher Scientific).

### 4.4. Quantitative PCR (qPCR) for Expression Analysis

MiRNA expression levels were analyzed by qPCR using TaqMan MiRNA Assays (Thermo Fisher Scientific): miR-124-3p (Assay ID: 001182), miR-125b-5p (Assay ID: 000449), miR-127-5p (Assay ID: 002229), miR-129-5p (Assay ID: 000590), miR-132-3p (Assay ID: 000457), miR-137-3p (Assay ID: 001129), miR-148a-3p (Assay ID: 000470), miR-191-5p (Assay ID: 002299), miR-193a-5p (Assay ID: 002281), miR-203a-3p (Assay ID: 000507), miR-339-3p (Assay ID: 002184), and miR-375-3p (Assay ID: 000564). RNU48 (Assay ID: 001006) and RNU6 (Assay ID: 001093) expression levels were used as references. The mRNA levels of *ZEB1* and *ZEB2* genes were assessed by qPCR using SYBR Green/ROX qPCR Master Mix (Thermo Fisher Scientific) on a CFX96 Real-Time PCR Detection System (Bio-Rad) with the primers and PCR conditions from the article by You et al. [63]. *B2M* was used as a reference gene in the *ZEB1* and *ZEB2* expression study [64]. All reactions were performed in triplicate, and each assay included negative control reactions that lacked cDNA. Relative quantification according to the ΔΔCt-method [65,66] was used for data analysis. Considering expression level variability of reference genes and estimated errors, less than twofold changes (ΔΔCt ≤1) in miRNA or mRNA levels were considered as retentions.

### 4.5. Quantitative Methylation-Specific PCR (qMSP)

Bisulfite DNA conversion and qMSP were performed as previously reported [67,68,69]. Briefly, the Epitect Fast DNA bisulfite kit (Qiagen, Hilden, Germany) was used for bisulfite conversion of DNA. The qPCRmix-HS SYBR reagent kit (Evrogen, Moscow, Russia) and CFX96 Real-Time PCR Detection System (Bio-Rad) were used. The primers designed for qMSP are listed in Supplementary Table S2. The completeness of DNA conversion was determined using the control *ACTB* locus with oligonucleotides specific to the unconverted template [68]. The absence of a PCR product in the case of unconverted DNA was checked for each pair of primers. Commercial DNA preparation #G1471 (Promega, Madison, WI, USA) was used as a control for unmethylated alleles. Commercial DNA preparation #SD1131 (Thermo Fisher Scientific) was used as a positive control for 100% methylation. The obtained data on the methylation of the studied genes were processed using the methylation index (MI) as a criterion of the methylation level as described earlier [70]. The MI value was calculated for each sample by the Bio-Rad software algorithm in the qPCR Bio-Rad system.

### 4.6. Overall Survival Markers

For most patients, overall survival data were tracked for more than 10 years. For the patient survival analysis depending on methylation levels of miRNA genes, the Kaplan–Meier curves, Fisher’s exact test, Cox proportional hazards model, and log-rank test were applied. To determine clinically significant parameters, such as the sensitivity (Sn) and specificity (Sp), the odds ratio (OR), and relative risk (RR), and to perform the ROC analysis, the MedCalc online calculator (https://www.medcalc.org/calc/diagnostic_test.php, accessed on 15 November 2021) was used.

### 4.7. Statistical Analysis

Statistical processing of the results was carried out using the package of statistical programs IBM SPSS Statistics 22, which included the determination of the median and interquartile range. The R software environment was used to build heatmaps and correlation matrices. To assess the significance of differences, the nonparametric Mann–Whitney U and multiple Kruskal–Wallis tests were used. Spearman’s correlation analysis was also applied. Differences were considered statistically significant at *p* ≤ 0.05. A Benjamini–Hochberg correction for multiple comparisons was performed and FDR (false discovery rate) values were calculated.

## Figures and Tables

**Table 1:** Comparison of the expression level of 12 miRNAs in nine peritoneal macroscopic metastases (PMM) and nine matched primary tumors.

miRNA	Tissue Samples	1st Quartile	Median	3rd Quartile
miR-124-3p	primary tumor	–2.46	0.54	3.28
	PMM	–2.60	–0.21	2.12
miR-127-5p	primary tumor	–3.16	–2.07	–0.24
	PMM	–5.70	–2.56	–0.97
miR-129-5p	primary tumor	–2.04	–1.76	–1.33
	PMM	–3.46	–2.37	–1.98
miR-132-3p	primary tumor	–1.79	–1.64	–0.57
	PMM	–5.54	–3.40	–0.16
miR-191-5p	primary tumor	–0.78	0.40	0.99
	PMM	–1.40	–0.29	0.03
miR-193a-5p	primary tumor	–3.01	–2.21	–1.61
	PMM	–4.06	–2.35	–1.98
miR-125b-5p	primary tumor	–4.33	–2.25	–0.61
	PMM	–4.53	–1.93	–0.81
miR-137-3p	primary tumor	–3.51	–2.21	0.74
	PMM	–4.28	–3.11	–0.56
miR-203a-3p	primary tumor	–3.43	–0.90	1.17
	PMM	–3.43	–1.21	–1.10
miR-375-3p	primary tumor	–5.03	–1.26	3.25
	PMM	–3.94	–2.35	–0.61
miR-148a-3p	primary tumor	–0.94	–0.70	0.42
	PMM	–2.91	–1.45	–0.38
miR-339-3p	primary tumor	–1.35	–1.21	0.43
	PMM	–1.74	–1.11	–0.95

Note: Expression levels are given relative to histologically normal tissues of the same patients; PMM—peritoneal macroscopic metastases.

**Table 2:** The relationship between methylation levels of miRNA genes and overall survival of OvCa patients according to the ROC analysis.

Gene	AUC	95% CI	Cutoff	The Significance Level (Area = 0.5)	Sn	Sp
*MIR107*	0.66	0.56–0.76	>18.89	0.004	53.7	75.9
*MIR124-1*	0.59	0.49–0.69	>11.24	0.124	53.7	68.5
*MIR124-2*	0.56	0.45–0.66	>7.19	0.345	78.1	44.4
*MIR124-3*	0.62	0.51–0.72	>34.97	0.043	41.5	79.6
*MIR125B-1*	0.64	0.53–0.73	>6.00	0.017	92.7	33.3
*MIR1258*	0.61	0.51–0.71	>7.96	0.051	78.1	48.2
*MIR127*	0.50	0.40–0.61	>5.29	0.959	73.2	37.0
*MIR129-2*	0.55	0.45–0.66	>36.87	0.385	53.7	66.7
*MIR130B*	0.68	0.57–0.77	>37.96	0.003	43.9	90.7
*MIR132*	0.55	0.45–0.66	≤11.25	0.374	65.9	53.7
*MIR137*	0.50	0.40–0.61	>0.34	0.991	85.4	3.7
*MIR148A*	0.52	0.41–0.62	>29.58	0.763	26.8	87.0
*MIR191*	0.58	0.47–0.68	>6.77	0.199	61.0	61.1
*MIR193A*	0.62	0.51–0.71	>32.33	0.052	65.9	59.3
*MIR203A*	0.54	0.43–0.64	>32.26	0.560	36.6	74.1
*MIR339*	0.57	0.47–0.68	>43.76	0.231	45.0	75.0
*MIR34B/C*	0.67	0.56–0.76	>12.79	0.003	65.9	68.5
*MIR375*	0.61	0.50–0.71	>14.95	0.064	56.1	64.8
*MIR9-1*	0.67	0.56–0.76	>55.43	0.004	46.3	85.2
*MIR9-3*	0.62	0.52–0.72	>21.89	0.046	61.0	70.4

Note: AUC—area under curve, Sn—sensitivity, Sp—specificity.

**Table 3:** The relationship between methylation levels of miRNA genes and overall survival of ovarian cancer patients according to the Fisher’s exact test and Cox proportional hazards model.

Gene, *MIR*	Frequency of Methylation Level Higher/Lower Cutoff Value for Living Patients	Frequency of Methylation Level Higher/Lower Cutoff Value for Deceased Patients	Fisher’s Exact Test, *p*	Cox Regr., *p*	Odds Ratio/ 95% CI	Relative Risk/ 95% CI
*107*	13/41	22/19	0.005	0.008	3.65/1.52–8.76	2.23/1.28–3.87
*124-1*	17/37	22/19	0.037	0.004	2.52/1.09–5.84	1.70/1.05–2.77
*124-2*	30/24	32/9	0.030	0.134	2.84/1.14–7.09	1.40/1.05–1.87
*124-3*	11/43	17/24	0.040	0.038	2.77/1.12–6.87	2.04/1.07–3.86
*125B-1*	36/18	38/3	0.003	0.006	6.33/1.72–23.3	1.39/1.13–1.71
*1258*	28/26	32/9	0.010	0.306	3.30/1.33–8.22	1.51/1.11–2.04
*127*	34/20	30/11	0.378	0.178	1.60/0.66–3.89	1.16/0.88–1.53
*129-2*	18/36	22/19	0.060	0.124	2.32/1.00–5.34	1.61/1.00–2.58
*130B*	5/49	18/23	0.0002	0.003	7.67/2.53–23.2	4.74/1.92–11.71
*132*	25/29	27/14	0.065	0.645	2.24/0.97–5.17	1.42/0.99–2.04
*137*	52/2	35/6	0.072	0.528	0.22/0.04–1.18	0.89/0.77–1.02
*148A*	7/47	11/30	0.115	0.020	2.46/0.86–7.05	2.07/0.88–4.87
*191*	21/33	25/16	0.040	0.037	2.46/1.07–5.65	1.57/1.04–2.37
*193A*	22/32	27/14	0.022	0.063	2.81/1.21–6.52	1.62/1.09–2.39
*203A*	14/40	15/26	0.273	0.416	1.65/0.68–3.97	1.41/0.77–2.58
*339*	15/39	19/22	0.084	0.188	2.25/0.95–5.28	1.67/0.97–2.87
*34B/C*	17/37	27/14	0.002	0.141	4.20/1.77–9.96	2.09/1.33–3.28
*375*	19/35	23/18	0.060	0.077	2.35/1.02–5.41	1.59/1.01–2.51
*9-1*	8/46	19/22	0.001	0.017	4.97/1.88–13.1	3.13/1.52–6.42
*9-3*	16/38	25/16	0.003	0.025	3.71/1.57–8.75	2.06/1.28–3.32

**Table 4:** Summary of clinical data for the sample set examined in the study.

Clinical and Histological Characteristics		*N* = 102	With PMM *N* = 30
Histological type	Borderline serous adenocarcinoma	6	0
	High-grade serous adenocarcinoma	73	25
	Low-grade serous adenocarcinoma	7	0
	Endometrioid adenocarcinoma	9	4
	Clear cell adenocarcinoma	2	0
	Mucinous adenocarcinoma	2	1
	Mixed epithelial tumors	2	0
	Undifferentiated carcinoma	1	0
Stage	I	12	1
	II	17	4
	III	67	24
	IV	6	1
Grade	G1	9	1
	G2	27	11
	G3	60	18
	Grade Unknown	6	0
Size	T1	12	1
	T2	18	4
	T3	72	25
Peritoneal metastases	Absent	72	0
	Present	30	30
Lymph node metastases	N0	81	30
	N1	21	0
Distant metastases	M0	96	29
	M1	6	1

Note: Data are given for the whole set of 102 primary tumors (left column) and separately for a subset of 30 primary tumors with peritoneal macroscopic metastases (PMM, right column).

## Data Availability

The data presented in this study are available on reasonable request from the corresponding author.

## Supplementary Materials

The following are available online at www.mdpi.com/article/10.3390/ijms23031300/s1.
