# Supplementary material for: Aberrant Methylation of 20 miRNA Genes Specifically Involved in Various Steps of Ovarian Carcinoma Spread: From Primary Tumors to Peritoneal Macroscopic Metastases
Source: Int J Mol Sci. 2022 Jan 24;23(3):1300. doi: 10.3390/ijms23031300 (PMC8835734; doi:10.3390/ijms23031300)
Supplement: Supplementary file 1 [file ijms-23-01300-s001.zip › Supplementary Table S2.pdf]

Supplementary Table S2. Primers and PCR conditions in the quantitative methylation-specific PCR (qMSP) study.

Gene	Sequences	T_{ann}, °C	Product, bp	Ref.
MIR124-1	MF: TAGGAAAAAGTTTGGATGCGAAA<br>MR: AAAAATAAAAATAAAAAACGCCGAA	55	147	[1]
	UF: GGAAAAAGTTTGGATGTGAAAGGATGG<br>UR: CCACCACAACTCCCCCAATCACA	61	98	
MIR124-2	MF: GGTTATGACGGAGAATATGTAATAGCG<br>MR: CAATTAATTCGCCCTAAATCCTTTC	56	90	[1]
	UF: AGGTGTGTGTTGTAAATGGTATGGAG<br>UR: CCCAACTCCTATCTCTACTCATCTCTA	54	137	
MIR124-3	MF: GATAGTATAGTCGGTTGAGCGTAGCGT<br>MR: CCTCAAAACTAAAACGAACGACGAAC	58	152	[1]
	UF: TAGTTGGTTGAGTGTAGTGTTTTTG<br>UR: CAAAACTAAAACAAACAACAAACATC	52	142	
MIR125B-1	MF: CGTTTTTTATTGAAATATTTCGTTTAG<br>MR: CGAAACCCGCACAACCTCCT	55	98	[1]
	UF: TTTTTGTTTTTTGTTTTTTATTGAAATA<br>UR: CAAAACCCACACAACCTCCTATAAC	53	109	
MIR127	MF: GTTTTGCGATGATGTTGAAGCGTTT<br>MR: CGCCAAACCGTAAAATTCCTAAACTT	60	181	[1]
	UF: GGGGTTTTGTGATGATGTTGAAGTGT<br>UR: CCACCAAACCATAAAATTCCTAAACTT	58	185	
MIR129-2	MF: GATTTTAGTTCGTATTAATGAGTTGGCGGTTTC<br>MR: CCGACTACAAAATCGCGAATCTCTAAAC	61	187	[1]
	UF: GATGATTTTAGTTTGTATTAATGAGTTGGTG<br>UR: CAACTACAAAATCACAAATCTCTAAACAA	55	189	
MIR132	MF2: TTTTTCGCGGTTTTGACGTTAGTTC<br>MR2:CGCCCCCGCCTCCTTCTACTC	62	213	[1]
	UF2: TGTTTTTTGTGGTTTTGATGTTAGT<br>UR2: CACCCCCACCTCCTTCTACTC	54	216	
MIR137	MF: GGTTTTTTGATTTTTTTCGGTGACG<br>MR: CCGCTAATACTCTCCTCGACTACGC	58	100	[1]
	UF: GGTTTTTTGATTTTTTTTGGTGATGG<br>UR: CCCCCCTACCACTAATACTCTCCTCAA	58	108	
MIR148A	MF: GTCGTTTTATTTTAGGGGTTTTTTTC<br>MR: CATTCCTAATCGAACCCTCACCT	55	205	[1]
	UF: GAATAGAGTATTGGGAATGGTTGG<br>UR: CATCTAAAAAACTAAAAACAAAAACAC	52	182	
MIR191	MF: CGTTGGTAGCGTTATCGGATAAA<br>MR: CCCACGAAACCTAAACCACTAATAC	55	162	[1]
	UF: GGTGTTTTTGAGGTGGATGTGTG<br>UR: CCACAAAACCTAAACCACTAATACAAT	55	122	
MIR193A	MF2: TTGGAGTTCGCGATTCGAGGTC<br>MR2: CTCATCTCGCCCGCAAAAACC	60	198	[1]
	UF2: GAGGGTTGGGTTTGGAGTTTGTGA<br>UR2:TAATCCAACACCCTCATCTCACC	60	221	

MIR203A	MF: TTTCGGGTCGTGGAGGATTAGTC<br>MR: ACTCCGAACGACGATAACCAACG	58	160	[1]
	UF: GTGGAGGATTAGTTGTGGGATTTAT<br>UR: CCAACACAACAACACCTTTTATACAA	54	134	
MIR339	MF: TGGTAGGAAGCGTTTTGTGTTTCGTA<br>MR: CATAATCCCGACGCAAACACTAAAAC	60	134	[1]
	UF: TGTAGGGGTGGTAGGAAGTGTTTTGTG<br>UR: CAAACTAACCACTCCATAATCCCAACAC	60	156	
MIR375	MF: CGTCGTTATCGTTATCGTTATTTTAATC<br>MR: AATTTCTATTCTAAACCACGACCCC	55	205	[1]
	UF: TTGTGTGTTGTTTTAGGGGAGATTTG<br>UR: ATAACTTACCCAAAACCATAAAAATCA	55	232	
MIR34B/C	MF: TTTAGTTACGCGTGTTGTGC<br>MR: ACTACAACTCCCGAACGATC	57	189	[2]
	UF: TGGTTTAGTTATGTGTGTTGTGT<br>UR: CAACTACAACTCCCAAACAATCC	57	190	
MIR9-3	MF: GAGGTAGGTCGGTAGCGTCGGTG<br>MR: ACCTAAACGAACGCCGTACCCG	60	197	[2]
	UF: AGAAATGTGTTGGGAGGGTGAGG<br>UR: ACCACTACCACACCATAAACTCCACAT	58	196	
MIR9-1	MF: TTTTATTTTCGTTGACGGGC<br>MR: CCCGCCTCCTAACTACTATCG	56	120	[3]
	UF: TTTTTTTATTTTTGTTGATGGGT<br>UR: CCCACCTCCTAACTACTATCACC	56	120	
MIR107	MF: TGTGTAGTAGTTCGTTTATAGC<br>MR: GACTCTACGACTACTAAATCG	55	220	[4]
	UF: TGTGTAGTAGTTTGTTTATAGTG<br>UR: CCAACTCTACAACTACTAAATC	55	220	
MIR130B	MF: AAAGATGGAGTCGGTAGGC<br>MR: AAACGCGAAAAATTAAACGA	56	109	[4]
	UF: GTTAAAGATGGAGTTGGTAGGT<br>UR: AAACACAAAAAATTAAACAAAAA	55	112	
MIR1258	MF: AGGTCGTGGAAGTTATAGGC<br>MR: CGAACCTACACCTAAACGC	57	126	[4]
	UF: ATTAGGTTGTGGAAGTTATAGGT<br>UR: AACAAACCTACACCTAAACACA	56	126	

Note: MF – forward primer for methylated allele; MR – reverse primer for methylated allele; UF – forward primer for unmethylated allele; UR – reverse primer for unmethylated allele.

References

1. Loginov, V.I.; Pronina, I.V.; Burdennyy, A.M.; Filippova, E.A.; Kazubskaya, T.P.; Kushlinsky, D.N.; Utkin, D.O.; Khodyrev, D.S.; Kushlinskii, N.E.; Dmitriev, A.A.; et al. Novel miRNA genes deregulated by aberrant methylation in ovarian carcinoma are involved in metastasis. *Gene* **2018**, *662*, 28-36, doi:10.1016/j.gene.2018.04.005.

2. Pronina, I.V.; Loginov, V.I.; Burdennyy, A.M.; Fridman, M.V.; Senchenko, V.N.; Kazubskaya, T.P.; Kushlinskii, N.E.; Dmitriev, A.A.; Braga, E.A. DNA methylation contributes

to deregulation of 12 cancer-associated microRNAs and breast cancer progression. *Gene* **2017**, *604*, 1-8, doi:10.1016/j.gene.2016.12.018.
3. Lujambio, A.; Calin, G.A.; Villanueva, A.; Ropero, S.; Sanchez-Cespedes, M.; Blanco, D.; Montuenga, L.M.; Rossi, S.; Nicoloso, M.S.; Faller, W.J.; et al. A microRNA DNA methylation signature for human cancer metastasis. *Proceedings of the National Academy of Sciences of the United States of America* **2008**, *105*, 13556-13561, doi:10.1073/pnas.0803055105.
4. Braga, E.A.; Loginov, V.I.; Burdennyi, A.M.; Filippova, E.A.; Pronina, I.V.; Kurevlev, S.V.; Kazubskaya, T.P.; Kushlinskii, D.N.; Utkin, D.O.; Ermilova, V.D.; et al. Five Hypermethylated MicroRNA Genes as Potential Markers of Ovarian Cancer. *Bulletin of experimental biology and medicine* **2018**, *164*, 351-355, doi:10.1007/s10517-018-3988-y.
