# Supplementary material for: Aberrant Methylation of 20 miRNA Genes Specifically Involved in Various Steps of Ovarian Carcinoma Spread: From Primary Tumors to Peritoneal Macroscopic Metastases
Source: Int J Mol Sci. 2022 Jan 24;23(3):1300. doi: 10.3390/ijms23031300 (PMC8835734; doi:10.3390/ijms23031300)
Supplement: Supplementary file 1 [file ijms-23-01300-s001.zip › Supplementary Table S1.pdf]

Supplementary Table S1. Clinical and histological characteristics for the 102 primary ovarian tumors examined in the study.

Number	Age	Stage	TNM	Histological type	Grade	Tumors with PMM	Twelve miRNA level	mRNA *ZEB1*/ *ZEB2*
1	56	III	T3N0M0	HGSC	3			
2	68	IV	T3N0M1	Clear cell adenocarcinoma	3			
3	25	III	T3N0M0	HGSC	3			
4	42	IIIc	T3cN1M0	HGSC	1			
5	42	III	T3N0M0	HGSC	3			
6	63	II	T2N0M0	HGSC	2			
7	60	III	T3N0M0	HGSC	3			
8	66	III	T3N0M0	HGSC	3			
9	51	III	T3N0M0	HGSC	3			
10	36	III	T3N0M0	HGSC	3			
11	63	I	T1N0M0	Mucinous adenocarcinoma	1			
12	62	IIIc	T3cN1M0	HGSC	3			
13	50	III	T3N0M0	HGSC	3			
14	56	IV	T2N1M1	HGSC	3			
15	32	III	T3N0M0	HGSC	3			
16	69	II	T2N0M0	Endometrioid adenocarcinoma	2			
17	74	I	T1N0M0	Mixed epithelial tumors	1			
18	39	IIIc	T3cN1M0	HGSC	3			
19	50	I	T1N0M0	LGSC	2			
20	62	III	T3N0M0	HGSC	3			

21	76	II	T2N0M0	Endometrioid adenocarcinoma	2			
22	53	IV	T3N1M1	HGSC	2			
23	55	IIIc	T3cN1M0	HGSC	3			
24	62	III	T3N0M0	HGSC	3			
25	52	I	T1N0M0	LGSC	1		+	
26	46	I	T1N0M0	LGSC	1		+	+
27	58	IIIc	T3cN1M0	HGSC	3		+	
28	31	IIIc	T3cN1M0	HGSC	3			
29	52	IIIc	T3cN1M0	HGSC	3			
30	48	III	T3N0M0	Endometrioid adenocarcinoma	3		+	+
31	47	II	T2N0M0	LGSC	1			
32	72	II	T2N0M0	HGSC	2		+	+
33	67	IV	T3cN1M1	HGSC	3		+	+
34	52	I	T1N0M0	LGSC	1		+	
35	57	IIIc	T3cN0M0	HGSC	3		+	+
36	51	IIIc	T3cN1M0	HGSC	3			
37	59	III	T3N0M0	HGSC	3		+	+
38	14	IIIc	T3cN1M0	HGSC	3		+	+
39	44	IIIc	T3cN1M0	Mixed epithelial tumors	2		+	+
40	69	IIIc	T3cN0M0	HGSC	3		+	
41	75	IIIc	T3cN1M0	HGSC	3			
42	75	IIIc	T3cN1M0	HGSC	3			
43	75	IIIc	T3cN1M0	HGSC	3			
44	35	III	T3N0M0	Borderline serous adenocarcinoma	nd		+	+
45	65	I	T1N0M0	Endometrioid adenocarcinoma	2		+	+

46	24	III	T3N0M0	Borderline serous adenocarcinoma	nd			
47	56	IIIc	T3cN1M0	HGSC	2		+	+
48	39	III	T3N0M0	Borderline serous adenocarcinoma	nd		+	+
49	69	II	T2N0M0	Clear cell adenocarcinoma	3		+	
50	78	I	T1N0M0	Endometrioid adenocarcinoma	2		+	+
51	46	II	T2N0M0	HGSC	3		+	+
52	58	IIIc	T3cN0M0	HGSC	3		+	+
53	28	II	T2N0M0	Borderline serous adenocarcinoma	nd		+	+
54	46	IIc	T2N0M0	HGSC	3		+	+
55	30	IIc	T2N0M0	HGSC	2		+	+
56	68	IIIc	T3cN0M0	HGSC	3		+	+
57	45	IIIc	T3cN1M0	HGSC	3		+	
58	63	IIIc	T3cN0M0	HGSC	2		+	+
59	66	IIIc	T3cN1M0	HGSC	3		+	+
60	53	IIIc	T3cN1M0	HGSC	3		+	+
61	54	II	T2N0M0	HGSC	1		+	+
62	75	IIIc	T3cN1M0	HGSC	3		+	+
63	64	IIIc	T3cN0M0	HGSC	3		+	
64	24	IIIb	T3bN0M0	Borderline serous adenocarcinoma	nd		+	+
65	44	II	T2N0M0	HGSC	3		+	+
66	63	IIc	T2cN0M0	HGSC	2		+	
67	77	IV	T3cN0M1	Undifferentiated carcinoma	3		+	
68	32	IIIc	T3cN0M0	HGSC	2	+		
69	76	I	T1N0M0	Borderline serous adenocarcinoma	nd		+	
70	39	IIIc	T3cN0M0	HGSC	2	+		

71	60	II	T2N0M0	HGSC	2	+		
72	50	IIIc	T3cN0M0	HGSC	3	+		
73	60	IIIc	T3cN0M0	HGSC	3	+		
74	53	IIIc	T3cN0M0	HGSC	3	+	+	
75	47	IIIc	T3cN0M0	HGSC	3	+		
76	59	I	T1N0M0	HGSC	2	+		
77	75	I	T1N0M0	LGSC	2		+	
78	44	III	T3N0M0	HGSC	2	+	+	
79	77	II	T2N0M0	Endometrioid adenocarcinoma	2	+	+	
80	57	I	T1N0M0	LGSC	2		+	
81	52	IIIc	T3cN0M0	HGSC	3			
82	58	IIIc	T3cN0M0	HGSC	2			
83	54	IIIc	T3cN0M0	HGSC	3	+		
84	55	IIIc	T3cN0M0	HGSC	3	+	+	
85	41	IIIc	T3cN0M0	HGSC	3	+	+	
86	42	IIIc	T3cN0M0	HGSC	3	+	+	
87	67	IIIc	T3cN0M0	Endometrioid adenocarcinoma	3	+	+	
88	54	IIIc	T3cN0M0	Mucinous adenocarcinoma	3	+	+	
89	60	IIc	T2cN0M0	Endometrioid adenocarcinoma	2	+	+	
90	49	IIIc	T3cN0M0	HGSC	2	+		
91	68	IIIc	T3cN0M0	HGSC	3	+		
92	74	IIIc	T3cN0M0	HGSC	3	+		
93	56	IIIc	T3cN0M0	HGSC	3	+		
94	49	IIIc	T3cN0M0	HGSC	3	+		
95	47	III	T3N0M0	HGSC	1	+		

96	60	IV	T3cN0M1	HGSC	3	+		
97	78	III	T3N0M0	HGSC	3	+		
98	58	III	T3N0M0	HGSC	2	+		
99	52	III	T3N0M0	HGSC	2	+		
100	57	II	T2N0M0	HGSC	2	+		
101	81	IIIc	T3cN0M0	Endometrioid adenocarcinoma	3	+		
102	50	IIIc	T3cN0M0	HGSC	3	+		

Note: HGSC – high-grade serous adenocarcinoma; LGSC – low-grade serous adenocarcinoma; nd – no data; 30 primary tumor samples studied with peritoneal macroscopic metastases (PMM) are marked by "+" and pale pink shading; 47 primary tumor samples examined in expression studies of 12 miRNAs are marked by "+" and pale green shading; 26 primary tumor samples examined in expression studies of *ZEB1* and *ZEB2* are marked by "+" and pale gray shading.
